# Comparative genomics in chicken and Pekin duck using FISH mapping and microarray analysis

**DOI:** 10.1186/1471-2164-10-357

**Published:** 2009-08-05

**Authors:** Benjamin M Skinner, Lindsay BW Robertson, Helen G Tempest, Elizabeth J Langley, Dimitris Ioannou, Katie E Fowler, Richard PMA Crooijmans, Anthony D Hall, Darren K Griffin, Martin Völker

**Affiliations:** 1 Department of Biosciences, University of Kent, Canterbury, CT2 7NJ, UK; 2 Institute of Cancer Research, Belmont, Surrey, SM2 5NG, UK; 3 Bridge Genoma, 1 St Thomas Street, London Bridge, London, SE1 9RY, UK; 4 Animal Breeding and Genomics Centre, Wageningen University, Marijkeweg 40, 6709 PG Wageningen, The Netherlands; 5 Cherry Valley Ltd, Rothwell, Market Rasen, Lincolnshire, LN7 6BJ, UK

## Abstract

**Background:**

The availability of the complete chicken (*Gallus gallus*) genome sequence as well as a large number of chicken probes for fluorescent *in-situ *hybridization (FISH) and microarray resources facilitate comparative genomic studies between chicken and other bird species. In a previous study, we provided a comprehensive cytogenetic map for the turkey (*Meleagris gallopavo*) and the first analysis of copy number variants (CNVs) in birds. Here, we extend this approach to the Pekin duck (*Anas platyrhynchos*), an obvious target for comparative genomic studies due to its agricultural importance and resistance to avian flu.

**Results:**

We provide a detailed molecular cytogenetic map of the duck genome through FISH assignment of 155 chicken clones. We identified one inter- and six intrachromosomal rearrangements between chicken and duck macrochromosomes and demonstrated conserved synteny among all microchromosomes analysed. Array comparative genomic hybridisation revealed 32 CNVs, of which 5 overlap previously designated "hotspot" regions between chicken and turkey.

**Conclusion:**

Our results suggest extensive conservation of avian genomes across 90 million years of evolution in both macro- and microchromosomes. The data on CNVs between chicken and duck extends previous analyses in chicken and turkey and supports the hypotheses that avian genomes contain fewer CNVs than mammalian genomes and that genomes of evolutionarily distant species share regions of copy number variation ("CNV hotspots"). Our results will expedite duck genomics, assist marker development and highlight areas of interest for future evolutionary and functional studies.

## Background

Comparative genomics allows the transfer of genomic information from a well-characterized species to another that is less well described. It can be applied at all levels from that of the chromosome to the genome sequence. However, despite the recent advances in sequencing technologies, the considerable effort involved in producing a genome sequence assembly is reflected by the small number of vertebrate genomes that have been sequenced to date. In birds, there is only one published genome sequence, that of the chicken [1], with the zebra finch genome due to be published soon.

Combining cross-species fluorescent in-situ hybridization (FISH) and microarray analysis using resources developed in the chicken provides a powerful tool for the identification of gross genomic rearrangements, gene gains/losses, copy number variants (CNVs) and gene order in other bird species. These techniques do not require sequence data for any species other than the reference (i.e. chicken). We have previously successfully used this approach for a genome wide comparison of chromosomal rearrangements and CNVs between chicken and turkey[2]. This revealed a strong conservation of genome structure over about 30 million years of evolution between chicken and turkey[3]. In particular, our results suggested that, when compared to mammalian genomes, bird genomes contain a low number of CNVs (i.e. polymorphisms in the number of copies of a DNA fragment 1 kb or larger[4], with the exception of insertions or deletions of transposable elements[5]). The latter finding indicates that patterns of CNVs in bird genomes mirror the low number of chromosomal rearrangements in this phylogenetic group[2,6].

Following on from the turkey, the Pekin duck (*Anas platyrhynchos*, APL) is the next obvious target among domestic birds for detailed genomic studies due to its agricultural importance, with worldwide duck consumption being between 4 and 5% of the total poultry market[7]. Duck is also an important target for immunological studies because of its resistance to avian influenza[8]. Despite this, genomic information about the duck is limited to a few linkage and physical mapping studies. Huang *et al*. [9] produced a preliminary genetic map based on 240 microsatellite loci and assigned 11 out of 19 linkage groups to ten duck (APL) chromosomes by FISH mapping of 28 BACs. Cross-species chromosome painting and G-banding studies [10-12] have suggested one interchromosomal difference between the chicken and duck karyotypes – the ancestral chromosomes 4 and 10, fused in the chicken lineage to give GGA4q and GGA4p respectively, remain separate in duck[6]. This interchromosomal rearrangement presumably explains the difference in diploid chromosome number between the two species, which is 2n = 78 in chicken and 2n = 80 in duck. FISH mapping of 57 chicken BACs revealed small intrachromosomal rearrangements in APL2, 7, 8 and Z and confirmed synteny for GGA9, 11, 13–15, 18 and 28 in the duck genome[13]. However, no molecular markers are available for the remaining microchromosomes, which are indistinguishable by conventional cytogenetics. It is also unclear which duck chromosome corresponds to GGA4p (ancestral chromosome 10). Thus, from a molecular cytogenetic standpoint, the duck genome is at present only partially defined, and given the low number of physical markers mapped by FISH, it is possible that hitherto undetected intrachromosomal rearrangements exist.

No data are currently available concerning CNVs in duck or indeed any other bird species than chicken and turkey. CNVs have been found to contribute significantly to normal and disease-related genetic and phenotypic variation in humans and other primates[5,14]. Studies of the evolutionary significance of CNVs have largely focused on primates and revealed numerous lineage-specific gene gains and losses and CNVs (e.g. [15-20]).

Our previous study of CNVs in chicken and turkey revealed a total of 16 CNVs[2]. Five of these CNVs appear to be shared in layer and broiler chickens, and in turkey, at regions dubbed "CNV hotspots" (i.e. genomic regions in which CNVs of approximately equal size were found in both chicken breeds and in turkey). Given that the contribution of CNVs to phenotypic variation is becoming increasingly clear, analyses of this kind of structural variation in organismic groups other than mammals are clearly needed.

Here, we present a detailed molecular cytogenetic map for the duck based on comparative FISH mapping of 155 chicken BACs, which revealed several hitherto undescribed intrachromosomal rearrangements. We also provide an analysis of CNVs in the duck genome by array comparative genomic hybridisation (array CGH) of duck DNA to a commercially available chicken whole-genome oligonucleotide tiling path microarray. The analysis of CNVs supports the hypotheses that bird genomes contain fewer CNVs than mammalian genomes and that some CNVs appear to be consistently shared across species, forming CNV hotspots.

## Results

### Comparative FISH mapping between chicken and duck

**Figure 1:**
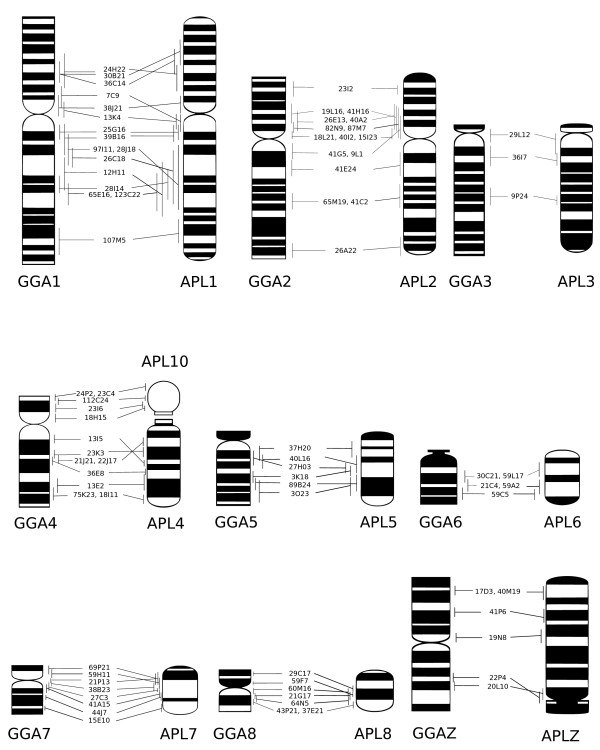
**Comparative map of chicken and duck chromosomes 1–8 and Z**. The G-banded ideograms of chicken (*Gallus gallus*; GGA) and duck (*Anas platyrhynchos; *APL) chromosomes 1–8 and Z are shown with the positions of all BACs successfully hybridized to both species as determined by FLpter measurements. Intrachromosomal rearrangements can be seen on GGA and APL1, 2, 4, 7, 8 and Z. GGA4p corresponds to APL10. Note the orientation of APLZ. Ideograms were prepared from[13,21]. Error bars represent one standard deviation.

**Figure 2:**
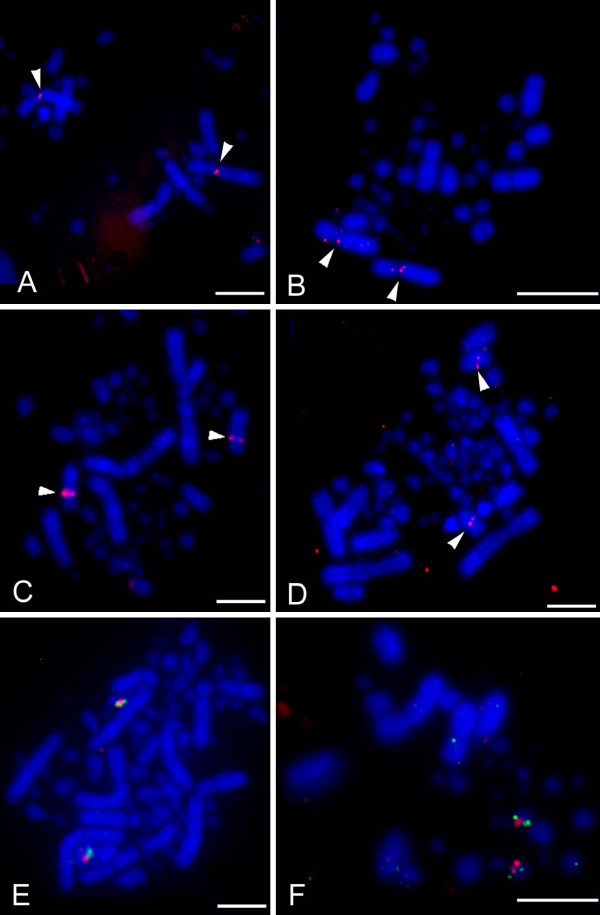
**Example FISH Results**. BAC WAG13K4 on A) chicken (*Gallus gallus*; GGA) chromosome 1p and B) duck (*Anas platyrhynchos*; APL) chromosome 1q. BAC WAG13I5 on C) chicken chromosome 4q and D) duck chromosome 4 demonstrating part of the evidence that led us to deduce a paracentric inversion. Conserved synteny among the microchromosomes was tested by dual color FISH. An example is shown for E) GGA15 and F) APL16 using BACs WAG10L1 and WAG93I1. Scale bars represent five microns.

Of 400 BACs that successfully hybridized to chicken metaphases, 155 (39%) could be visualized with confidence on duck chromosomes. These covered the majority of the karyotype i.e. APL1–29 and Z (except 26). Figure 1 shows the G-banded ideograms for GGA1–8 and Z and APL1–8 and Z[13,21], with the positions of the BACs mapped to these chromosomes; the full data are presented in Additional file 1. Figure 2 shows example FISH results. Only one interchromosomal difference was detected among the macrochromosomes, the retention of the ancestral chromosomes 4 and 10 in duck (which are fused in chicken).

FISH mapping suggested intrachromosomal rearrangements on GGA1, 2, 4, 7, 8, Z and APL1, 2, 4, 7, 8 and Z. BACs WAG24H22, WAG30B21 and WAG36C14 clearly showed a rearrangement on GGA1p and APL1p. The order of BACs was not completely inverted, suggesting that the underlying rearrangement may be a translocation rather than a paracentric inversion. BACs WAG7C9 and WAG13K4 (Figure 2A, B) mapped to GGA1p and APL1q, indicating a small pericentric inversion. Besides, some BACs mapping to GGA1q and APL1q suggested possible rearrangements on these chromosome arms; however, the high standard deviations in the FLpter measurements determined for these BACs in duck made it difficult to distinguish artefacts from real changes in marker order.

BACs WAG42G5 and WAG9L1 mapped to GGA2q and APL2p, providing clear evidence of a pericentric inversion. However, BAC WAG18G1, which mapped close to the centromere on GGA2p, also hybridized close to the centromere in APL2p. This demonstrated that the inversion involves only a small fraction of 2p.

BACs WAG13I5, WAG23K3, WAG21J21 AND WAG22J17 clearly demonstrated a paracentric inversion on GGA4q and APL4 (example image of WAG13I5 in Figure 2C, D).

The morphological differences between GGA7 and APL7 were reflected in a change in marker order involving BACs WAG69P21, WAG59H11 and WAG21P13. However, like in the rearrangement on GGA1p and APL1p, marker order was not completely inverted, indicating that this rearrangement may be more complex than a simple pericentric inversion. Similarly, our FISH mapping results did not provide clear evidence for a pericentric inversion causing the morphological differences between GGA8 and APL8.

Marker order on GGAZ and APLZ chromosome was largely conserved, with the possible exception of a small inversion involving BACs WAG22P4 and WAG20L10. Thus, it seems that the morphological differences between the metacentric GGAZ and the subtelocentric APLZ are due to neocentromere formation rather than a pericentric inversion.

Dual-color FISH experiments with BACs mapping to either end of a microchromosome were used to check for conserved synteny among the microchromosomes. This demonstrated conserved synteny for APL9, 11, 14–16, 19, 21, 27–29. The BACs successfully hybridized to the microchromosomes are listed in Additional file 2; example results are shown in Figure 2E, F.

FISH mapping of BACs WAG112C24 and WAG23I06 facilitated the identification of the duck orthologue of GGA4p. Chromosome area measurements suggested that this chromosome should be numbered as APL10. There were no major differences in size order between the remaining microchromosomes and their chicken orthologues.

### Copy number variation between chicken and duck

Hybridisation of genomic DNA from two female ducks to the Nimblegen chicken whole genome tiling path microarray revealed 32 CNVs, of which ten were seen in both individuals (CNVs marked with ^a ^in Table 1). Both gains and losses relative to chicken were seen (8 gains and 24 losses). The mean and median lengths of the detected CNVs were 281 kb and 50 kb respectively, ranging from 2.8 kb to 4.4 Mb. The CNV locations were compared with those previously found in turkey and chicken broilers and layers[2]. Six CNVs overlapped with CNVs identified in turkey, and of these five CNVs matched the five potential CNV 'hotspots' described by Griffin *et al*. [2] (bolded in Table 1). Three of the 'hotspot' CNVs were seen in both ducks. Known or predicted genes were found to be associated with 22 of the 32 CNVs (68.75%). In the ten cases where no genes were associated, all were losses, and all except one were found either near to the beginning or end of the chromosome (between 2 bp and 4 Mb of the sequence start or end according to Ensembl, http://www.ensembl.org/Gallus_gallus). The exception, CNV#14, covered a sequence gap, which likely contains centromeric repeats. Two of the CNVs, #7 and #9, were in regions that are potentially involved in rearrangements on GGA7 and 8 respectively.

**Table 1:** CNVs detected in duck (*Anas platyrhynchos*) relative to chicken reference DNA.

CNV	Chicken chromosome	Start (bp)	Stop (bp)	Size (kb)	Fold change c.f. Red Junglefowl	Comments
**1^ab^**	**1**	**140580435**	**140655038**	**74.603**	**-4.23**	**Novel genes**

2	3	107012500	108262500	1250	-1.43	Mitochondrial oxidoreductase (β-oxidation cycle)

**3^ab^**	**3**	**113237500**	**113651334**	**413.834**	**-1.73**	**39S ribosomal protein L19 (ribosomal subunit)**

4	3	113605240	113652668	47.428	-3.88	39S ribosomal protein L19 (ribosomal subunit)

**5^b^**	**4**	**88710224**	**89072982**	**362.758**	**-1.61**	**Histone demethylation protein**

6	6	37387597	37400184	12.587	-1.86	

7	7	352	5048	4.696	-2.66	

8	7	38350336	38380092	29.756	-2.85	N-acetyltransferase 5 (acetylation of amino-terminal methionine residues)

9^a^	8	102	35016	34.914	-3.54	

10	10	252	27706	27.454	-1.54	Novel genes

11	10	22517883	22537510	19.627	-2.26	

12^a^	11	202	15241	15.039	-8.50	

13	11	12500	62500	50	-1.41	Novel genes

14^a^	11	2675046	3190227	515.181	-2.76	Centromere

15^a^	12	12500	62500	50	-2.86	Senescent cell antigen-like-containing domain protein (Plays a role in modulating cell spreading and migration)

16	13	4932529	4935329	2.8	3.75	Teneurin 2 (transcription factor activity)

17^b^	13	18865302	18907544	42.242	-2.28	

18	15	10240354	10295196	54.842	2.29	Novel genes

**19^b^**	**16**	**352**	**432851**	**432.499**	**-1.43**	**Covers all GGA16 sequence available; MHC**

20	16	262500	287500	25	1.50	Predicted similar to MHC Rfp-Y class I alpha chain

21^a^	17	802976	867945	64.969	1.99	Neural proliferation differentiation and control protein

22	18	1812500	1837500	25	-1.46	Novel genes

23	19	3665182	3845095	179.913	1.48	Myosin regulatory light chain, cardiac muscle isoform

24	22	3912586	3935246	22.66	1.43	Similar to 2-oxoglutarate dehydrogenase (TCA cycle)

25	26	481	5060	4.579	-1.92	

26	26	4577801	4750398	172.597	1.62	Forkhead box protein; MyoD family inhibitor (transcriptional regulators)

27^a^	26	2911	837508	834.597	-1.79	Novel proteins

28^a^	28	2	12526	12.524	-7.87	

29	28	12500	62500	50	-2.48	Major vault protein (ribonucleoprotein complex)

30	E64	402	45047	44.645	-1.43	Predicted similar to ligase (DNA replication)

31	Z	2090459	6530135	4439.676	1.59	Synaptotagmin-4 (neurotransmitter secretion)

**32^ab^**	**Z**	**71512500**	**71762500**	**250**	**-5.09**	**Novel genes**

^a ^CNVs found in both individuals

^b ^CNVs also seen in turkey [2]

The 5 CNVs overlapping the 5 potential hotspots from [2] are bolded.

## Discussion

### Comparative cytogenetic map of the duck genome

Previous studies of avian genome evolution using cross-species FISH have suggested that gross genome structure is remarkably conserved among birds. Duck is no exception from this pattern; in fact it appears that the duck karyotype corresponds very closely to the putative ancestral avian karyotype [6]. The conservation of ancestral chromosomes 4 and 10, as APL4 and APL10 are consistent with both the previous studies on duck and with broader patterns of avian genome evolution[6]. These chromosomes appear intact in almost all birds, and ancestral chromosome 4 is seen intact in human chromosome 4 as well[6,13]. Our finding that only 39% of the BACs provided identifiable signals is attributable to the evolutionary distance between chicken and duck (90 million years); we achieved a 70% success rate mapping chicken BACs in turkey [2] (which diverged from chicken 30 million years ago), and our experience suggests that success rates of cross-species FISH with BACs rapidly decrease with increased evolutionary distance. This is also reflected by increased levels of non-specific background hybridisation from repetitive content, especially on the macrochromosomes, accounting for our wider error bars when assigning positions to BACs on these chromosomes.

In agreement with previous studies, no other interchromosomal rearrangements were detected between chicken and duck. However, our BAC mapping data are consistent with intrachromosomal rearrangements distinguishing chromosomes GGA1, 2, 4, 7, 8, Z and APL1, 2, 4, 7, 8 and Z, which confirms and expands on previous findings[13]. The detection of additional rearrangements on GGA1 and APL1 and GGA4q and APL4 was due to the much higher number of BACs hybridized in this study compared to previous studies. Likewise, higher density mapping demonstrated that the morphological differences between GGAZ and APLZ are probably due to the formation of a neocentromere rather than a pericentric inversion. This type of chromosomal rearrangement was previously reported in birds only for the red-legged partridge [23] and the Japanese quail[24]. However, despite the good coverage of the duck cytogenetic map presented here, it was not possible to determine unequivocally the nature of all chromosomal rearrangements observed between chicken and duck. It seems likely however that, in addition to peri- and paracentric inversions and neocentromere formation, translocations contributed to avian genome evolution. This conclusion is based on the order of BACs associated with rearrangements on GGA1, 7 and 8 and APL1, 7 and 8, which is not entirely consistent with the order expected if the rearrangements were inversions. Thus, it appears that while the available data from comparative FISH mapping suggest a relatively low frequency of intrachromosomal rearrangements in the evolution of bird genomes, the underlying processes may be more diverse than previously appreciated. Undoubtedly, the higher resolution afforded by genome sequencing projects such as that of the zebra finch will help to resolve this question.

The evolutionary direction of intrachromosomal changes could be determined for GGA8 and APL8 only. Comparison with the turkey map [2] suggested that APL8 likely represents the ancestral state; the order of BACs on turkey chromosome 10 (ancestral chromosome 8) and the morphology of this chromosome is the same as in duck, indicating that the rearrangement has occurred in the chicken lineage. Due to a lack of comparative data, it was not possible to determine the polarity of the remaining intrachromosomal rearrangements. If BAC positions are compared between this map and the turkey map (Additional file 1, [2]), the differences fit the patterns expected from the previously described inter- and intra-chromosomal rearrangements.

Among the rearrangements that we detected, the inversion observed in GGA4q and APL4 is of particular interest. Morphological differences in GGA4 have been described between different chicken breeds[25], and the ancestral bird chromosome 4 (corresponding to GGA4q and APL4, respectively) is also one of the chromosomes most prone to convergent independent fusions in birds (with ancestral chromosome 10) [6]. This contrasts with the conserved synteny of the ancestral bird chromosome 4 (corresponding to GGA4q and APL4, respectively) in humans. Together, the data suggest that rearrangements in chromosome 4 may be more common than has been suspected from previous comparative genomic studies, and analyzing them will prove valuable for understanding avian and other vertebrate genome evolution.

The present study extended the data previously available for conserved synteny among the microchromosomes. Fillon *et al*. [13]demonstrated conserved synteny for seven microchromosome pairs (APL9, 12, 14–16, 19, 29); here we demonstrate conserved synteny for ten microchromosome pairs (APL9, 11, 14–16, 19, 21, 27–29). The lack of detected rearrangements makes it reasonable to suggest that there are very few, if any, rearrangements among the remaining microchromosomes – including the as-yet unexamined smallest microchromosomes for which no markers exist. Indeed, no sequence data from the chicken genome has yet been assigned to these smallest chromosomes, and it is still unclear why, although there is a suggestion that there may be a cloning or sequencing bias against microchromosomal sequences[26]. Data on conserved microchromosomal synteny in other bird species are restricted to the Japanese quail (*Coturnix japonica*[27]) and the turkey[2]. Despite the paucity of data, the emerging picture is one of remarkable conservation among most avian species, with the exception of a few groups where large-scale interchromosomal rearrangements are common (such as the Falconiformes or Psittaciformes[28,29]).

The detailed cytogenetic map allowed for an improved definition of the duck karyotype. Chromosome banding and macrochromosome painting studies had previously shown orthology of APL1–9 and Z to GGA1–3, 4q, 5–9 and Z [10-12]. However, it was not known which duck chromosome corresponded to chicken chromosome 4p; Fillon *et al*. [13]suggested that this was approximately APL10–13. In this study we have used a combination of BAC mapping and chromosome area measurements which suggest that this chromosome should be numbered as APL10. Moreover we found no evidence for rearrangements among the microchromosomes. Accordingly, we propose that duck chromosomes be numbered as per chicken for 1–9; that APL10 corresponds to GGA4p; then GGA10 onwards correspond to APL11 onwards. The successful hybridization of at least one BAC from GGA1–28 (except 25) means markers are now available for APL1–29 (except 26) and Z. Taken together, these results enabled us to define unequivocally chromosomes APL1–29.

### Copy number variation between chicken and duck

The purpose of our array CGH experiments was to test two of our earlier hypotheses: (i) that birds show a reduced number of CNVs compared to mammals, (ii) that genomic CNV hotspots described previously in chicken and turkey [2] are found in the duck as well (indicating conservation over a large evolutionary distance). The successful hybridizations that we observed, despite approximately 90 million years of divergence between chicken and duck[31], extend avian cross-species microarray experiments from the sole previous study in turkey[2]. The present study revealed a total of 32 CNVs in the duck when compared to the chicken, which is substantially fewer than the 58 CNVs discovered in a comparison of human and chimpanzee[31], which diverged only 6–7 million years ago[32]. While this result supports the hypothesis that bird genomes show fewer CNVs than mammalian ones, it should also be noted that we found twice as many CNVs in duck as were found in the comparison of turkey and chicken (32 versus 16) [2]. Only ten of these CNVs were found in both duck specimens examined, indicating substantial intraspecific variation. These findings highlight the need for further studies with larger sample sizes and call for some caution when comparing the frequency of CNVs in birds and mammals.

The comparison of CNVs between chicken breeds and turkey revealed five tentative CNV hotspots by virtue of the fact that they contained CNVs of similar size in different chicken lines and in turkey[2]. Of these five hotspot regions, three contained CNVs in both ducks and two contained CNVs in one duck. The hotspot regions contain a number of novel genes including most of the available sequence for GGA16, covering the MHC loci. Moreover these findings lend support to our avian "CNV hotspot hypothesis" but, of course, need to be confirmed by analysing a wider number of species.

All copy number gains (in duck compared to chicken) were located in coding regions. Genes in regions of copy number gain relative to chicken included transcription factors, neural proliferation control and neurotransmitter activity, and a predicted MHC class I gene. This is consistent with previously described duplication of the MHC class I locus in the mallard duck, followed by subsequent inactivation of some of the extra gene copies[33].

Where copy number losses relative to chicken were detected, two possible explanations exist: a true copy number loss, or sequence divergence leading to lack of hybridization on the microarray. If such sequence divergence had occurred, however, the loss might be expected in both individuals. This was only seen for 9 CNVs; the remainder are more likely to be true polymorphic copy number differences. However, it is important to note that about two thirds of all apparent copy number losses were observed in coding regions; hence, the observed loss in hybridization efficiency is likely associated with functional consequences, regardless of whether it is due to copy number change or sequence divergence. Thus these results highlight genomic regions that are of particular interest for further functional and evolutionary studies.

It has been suggested that segmental duplications are correlated with CNVs, and facilitate chromosomal rearrangements, the lack of segmental duplications in birds therefore explaining the relative paucity of CNVs[1]. Due to the vastly different levels of resolution afforded by cytogenetic mapping and microarray analysis, it is difficult to directly correlate the results of these two methods. Nevertheless, it is interesting to note that two of the CNVs revealed in this paper appear to coincide with rearrangements detected from the cytogenetic mapping. These are CNVs #7 and #9, on chromosomes GGA7 and 8 respectively. Further studies are necessary to examine this link between chromosomal rearrangements and CNVs in more detail.

## Conclusion

The comparative cytogenetic map of the duck presented here highlights the extraordinary conservation seen among the genomes of many bird species, and how little structural genetic variation is readily apparent. The cytogenetic map will allow the transfer of genetic information directly from chicken to duck, expediting mapping studies in the duck and help to target marker development in duck through the prediction of new loci. The combination of area measurements and FISH mapping of chicken BACs allowed the identification of markers for chromosomes APL1–24 and 26–29 which will facilitate further mapping studies in the duck. Moreover, we have extended the analysis of CNVs in birds, providing further evidence that birds have low numbers of CNVs when compared to mammals and that bird genomes contain CNV hotspots. While overall we confirm the evolutionary conservation of bird genomes, the intrachromosomal differences and CNVs found highlight areas of future interest for evolutionary and functional studies.

## Methods

### Cell culture and chromosome preparation

All chromosome preparations were made from cultured cells derived from fertilized eggs. Chicken eggs were supplied by Hill Top Farm, Cambridgeshire, UK and Friday's Farm, Kent, UK. Duck eggs were provided by Cherry Valley Ltd, Market Rasen, UK. Fibroblast cultures were established from 5- to 7-day-old embryos. Chromosome preparation followed standard protocols[34,35]: mitostatic treatment with colcemid at a final concentration of 0.1 μg/ml for 1 h at 37°C, hypotonic treatment with 75 mM KCl for 15 min at 37°C and fixation with 3:1 methanol:acetic acid.

### Selection and preparation of BAC clones

400 BAC clones were selected from the Wageningen chicken BAC library [36] based on the position of markers on the chicken consensus linkage map[37]. The BACs were labeled by nick translation with biotin-16-dUTP or digoxigenin-11-dUTP (Roche) following standard protocols. At least one BAC was available for GGA1–28 (except 25) and Z. The 155 BACs successfully hybridized to both chicken and duck are detailed in Additional files 1 and 2.

### Fluorescent *in-situ *hybridisation (FISH)

Slides with metaphase preparations were aged for one hour at 70°C on a hotplate then treated with 4 mg/ml RNase A for one hour at 37°C. The chromosomes were denatured for 1 minute 30 seconds in 70% formamide in 2×SSC at 70°C. BACs were applied to slides and sealed under coverslips with rubber cement. Hybridization was carried out in a humidified chamber for 72 hours at 37°C. Following post-hybridization washes (40% or 30% formamide in 2×SSC for 20 minutes; 1 minute in 2×SSC/0.1% Igepal at RT; 15 minutes in 4×SSC/0.05% Tween 20 at RT; 25 minutes in 4×SSC/0.05% Tween 20/2% BSA at RT), probes were detected with 1:200 streptavidin-Cy3 (Amersham), plus 1:200 FITC-anti-digoxigenin (Amersham) for dual-color experiments, in 4×SSC, 0.05% Igepal, 1.25% BSA for 35 minutes. Slides were washed in 4×SSC, 0.05% Igepal for 3 × 5 minutes then counterstained using Vectashield with DAPI (Vector Labs).

Dual-color FISH was used to determine whether there was conserved synteny among the microchromosomes, selecting BACs that were as close as possible to the ends of the chromosomes. For GGA11, only one hybridizing BAC in duck was available.

### Image capturing and analysis

Slides were analyzed on an Olympus BX-61 epifluorescence microscope equipped with a cooled CCD camera and appropriate filters. Images were captured using SmartCapture 3 (Digital Scientific UK). The signal positions were measured as the fractional length from the p-terminus (FLpter[38]). FLpter and area measurements were carried out in ImageJ[39]. The area of duck chromosomes was determined as a ratio of the easily recognized chromosome 5, as per[40]. For area measurements, 10 metaphases were measured per chromosome; the numbers of metaphases used for FLpter measurements are given in Additional file 1.

### Array CGH

The NimbleGen chicken whole-genome tiling array (Catalogue Number/Design Name B3791001-00-01, galGal3 WG CGH – Roche NimbleGen, Milton Keynes, UK) was used for the array CGH experiments. It contains 385,000 50-mer oligonucleotides with an average spacing of 2,586 base pairs (source – UCSC, build – galGal3) and was interrogated with duck whole genomic DNA extracted from blood from two female ducks using a DNeasy Animal Blood and Tissue kit (Qiagen, #69504); the reference (Red Jungle Fowl) DNA, from the same animal used in the chicken genome sequencing project, was kindly provided by Dr Hans Cheng (Michigan State University). Labeling of genomic DNA and hybridization to the Roche NimbleGen array were performed by the company (Roche NimbleGen) and used random priming to incorporate modified nucleotides by either amino-allyl or direct linkage to either of the two dyes used (Cy3 and Cy5). All of the hybridizations in this experiment used two dyes per slide (Cy3 and Cy5). Red Jungle Fowl reference DNA (Cy5) was co-hybridized with duck test DNA (Cy3).

CGH analysis proceeded in three stages, normalization, window averaging and segmentation. After combining the signal intensity and genomic coordinate information, the Cy3 and Cy5 signal intensities were normalized to one another using Qspline normalization[41]. Qspline is a robust non-linear method for normalization using array signal distribution analysis and cubic splines. Once normalized, the data was prepared for DNA segmentation analysis. This included a window averaging step, where the probes that fall into a defined base pair window size (25 kb) are averaged, using the Tukey's biweight mean[42]. The Tukey's biweight method yields a robust weighted mean that is relatively insensitive to outliers, even when extreme. A new position was assigned to this average, which is the midpoint of the window. Segmentation was also performed on unaveraged data to permit smaller segments than the window size to be detected. The circular binary segmentation algorithm [43] was used to segment the averaged log2 ratio data. DNA segments were called by attempting to break the segments into sub-segments by looking at the t-statistic of the means. Permutations (n = 1000) were used to provide the reference distribution. If the resulting p-value was below the threshold (default of p = 0.01), then a breakpoint was called. A pruning step was used to remove spurious segments, rejecting segments where the standard deviation of the means was not sufficiently different. By default, a cut off of 1.5 standard deviations was used. CNVs were called for segments in which the log2 ratio was greater than | ± 0.5|. Where overlapping CNVs were detected in window averaged and unaveraged data, the data were considered to represent a single CNV.

## Authors' contributions

BMS performed probe preparation, hybridisations, microscopy and data analysis and prepared the manuscript. LBWR, HGT, EJL, DI and KEF assisted with probe preparation and microscopy. RPMAC provided the BAC clones. ADH and DKG conceived and designed the study. DKG was the PI on the BBSRC grant that funded the project and supervisor of BMS, LBWR, HGT, EJL, DI, KEF and MV. DKG critically revised the manuscript. MV performed hybridisations, data analysis and prepared the manuscript. All authors read and approved the final manuscript.

## Supplementary Material

Additional file 1 **Chicken (*Gallus gallus*; GGA) BACs successfully hybridized to duck (*Anas platyrhynchos*; APL) macrochromosomes**. BACs with these markers successfully hybridized to duck chromosomes. FLpter represents Fractional Length from the p terminus[38]; SD represents standard deviation. For reference, our previous data on turkey (*Meleagris gallopavo*, MGA) [2] is also included. Click here for file

Additional file 2 **BACs on duck (*Anas platyrhynchos*; APL) microchromosomes**. BACs with the named markers successfully hybridized to duck chromosomes. Duck chromosome number after APL10 was assigned as chicken (GGA) chromosome number plus one. The orthologous turkey (*Meleagris gallopavo*, MGA) chromosome (from [2]) is also listed. Click here for file

## References

[B1] Hillier LW Miller W Birney E Warren W Hardison RC Ponting CP Bork P Burt DW Groenen MA Delany ME Dodgson JB Chinwalla AT Cliften PF Clifton SW Delehaunty KD Fronick C Fulton RS Graves TA Kremitzki C Layman D Magrini V McPherson JD Miner TL Minx P Nash WE Nhan MN Nelson JO Oddy LG Pohl CS Randall-Maher J Smith SM Wallis JW Yang SP Romanov MN Rondelli CM Paton B Smith J Morrice D Daniels L Tempest HG Robertson L Masabanda JS Griffin DK Vignal A Fillon V Jacobbson L Kerje S Andersson L Crooijmans RP Aerts J Poel JJ van der Ellegren H Caldwell RB Hubbard SJ Grafham DV Kierzek AM McLaren SR Overton IM Arakawa H Beattie KJ Bezzubov Y Boardman PE Bonfield JK Croning MD Davies RM Francis MD Humphray SJ Scott CE Taylor RG Tickle C Brown WR Rogers J Buerstedde JM Wilson SA Stubbs L Ovcharenko I Gordon L Lucas S Miller MM Inoko H Shiina T Kaufman J Salomonsen J Skjoedt K Wong GK Wang J Liu B Yu J Yang H Nefedov M Koriabine M Dejong PJ Goodstadt L Webber C Dickens NJ Letunic I Suyama M Torrents D von Mering C Zdobnov EM Sequence and comparative analysis of the chicken genome provide unique perspectives on vertebrate evolution Nature 2004 432 7018 695 716 10.1038/nature03154 15592404

[B2] Griffin DK Robertson LB Tempest HG Vignal A Fillon V Crooijmans RP Groenen MA Deryusheva S Gaginskaya E Carre W Waddington D Talbot R Volker M Masabanda JS Burt DW Whole genome comparative studies between chicken and turkey and their implications for avian genome evolution BMC Genomics 2008 9 168 10.1186/1471-2164-9-168 18410676 PMC2375447

[B3] Pereira SL Baker AJ A molecular timescale for galliform birds accounting for uncertainty in time estimates and heterogeneity of rates of DNA substitutions across lineages and sites Mol Phylogenet Evol 2006 38 2 499 509 10.1016/j.ympev.2005.07.007 16112881

[B4] Feuk L Marshall CR Wintle RF Scherer SW Structural variants: changing the landscape of chromosomes and design of disease studies Human Molecular Genetics 2006 15 Spec No 1 R57 66 10.1093/hmg/ddl057 16651370

[B5] Freeman JL Perry GH Feuk L Redon R McCarroll SA Altshuler DM Aburatani H Jones KW Tyler-Smith C Hurles ME Carter NP Scherer SW Lee C Copy number variation: new insights in genome diversity Genome Res 2006 16 8 949 961 10.1101/gr.3677206 16809666

[B6] Griffin DK Robertson LB Tempest HG Skinner BM The evolution of the avian genome as revealed by comparative molecular cytogenetics Cytogenet Genome Res 2007 117 1–4 64 77 10.1159/000103166 17675846

[B7] Food and Agriculture Organisation of the United Nations http://faostat.fao.org/

[B8] Munster VJ Veen J Olsen B Vogel R Osterhaus AD Fouchier RA Towards improved influenza A virus surveillance in migrating birds Vaccine 2006 24 44–46 6729 6733 10.1016/j.vaccine.2006.05.060 16806601

[B9] Huang Y Zhao Y Haley CS Hu S Hao J Wu C Li N A Genetic and Cytogenetic Map for the Duck (Anas platyrhynchos) Genetics 2006 173 1 287 296 10.1534/genetics.105.053256 16510785 PMC1461431

[B10] Denjean B Ducos A Darre A Pinton A Seguela A Berland H Blanc M Fillon V Darre R Caryotype des canards commun (Anas platyrhynchos), Barbarie (Cairina moschata) et de leur hybride Revue Med Vet 1997 148 8–9 695 704

[B11] Schmid M Nanda I Guttenbach M Steinlein C Hoehn M Schartl M Haaf T Weigend S Fries R Buerstedde JM Wimmers K Burt DW Smith J A'Hara S Law A Griffin DK Bumstead N Kaufman J Thomson PA Burke T Groenen MA Crooijmans RP Vignal A Fillon V Morisson M Pitel F Tixier-Boichard M Ladjali-Mohammedi K Hillel J Maki-Tanila A Cheng HH Delany ME Burnside J Mizuno S First report on chicken genes and chromosomes 2000 Cytogenet Cell Genet 2000 90 3–4 169 218 10.1159/000056772 11124517

[B12] Schmid M Nanda I Hoehn H Schartl M Haaf T Buerstedde JM Arakawa H Caldwell RB Weigend S Burt DW Smith J Griffin DK Masabanda JS Groenen MA Crooijmans RP Vignal A Fillon V Morisson M Pitel F Vignoles M Garrigues A Gellin J Rodionov AV Galkina SA Lukina NA Ben-Ari G Blum S Hillel J Twito T Lavi U David L Feldman MW Delany ME Conley CA Fowler VM Hedges SB Godbout R Katyal S Smith C Hudson Q Sinclair A Mizuno S Second report on chicken genes and chromosomes 2005 Cytogenet Genome Res 2005 109 4 415 479 10.1159/000084205 15905640

[B13] Fillon V Vignoles M Crooijmans RP Groenen MA Zoorob R Vignal A FISH mapping of 57 BAC clones reveals strong conservation of synteny between Galliformes and Anseriformes Anim Genet 2007 38 3 303 307 10.1111/j.1365-2052.2007.01578.x 17539975

[B14] Emanuel BS Saitta SC From microscopes to microarrays: dissecting recurrent chromosomal rearrangements Nat Rev Genet 2007 8 11 869 883 10.1038/nrg2136 17943194 PMC2858421

[B15] Bailey JA Eichler EE Primate segmental duplications: crucibles of evolution, diversity and disease Nat Rev Genet 2006 7 7 552 564 10.1038/nrg1895 16770338

[B16] Dumas L Kim YH Karimpour-Fard A Cox M Hopkins J Pollack JR Sikela JM Gene copy number variation spanning 60 million years of human and primate evolution Genome Res 2007 17 9 1266 1277 10.1101/gr.6557307 17666543 PMC1950895

[B17] Fortna A Kim Y MacLaren E Marshall K Hahn G Meltesen L Brenton M Hink R Burgers S Hernandez-Boussard T Lineage-specific gene duplication and loss in human and great ape evolution PLoS Biol 2004 2 7 E207 10.1371/journal.pbio.0020207 15252450 PMC449870

[B18] Locke DP Segraves R Carbone L Archidiacono N Albertson DG Pinkel D Eichler EE Large-scale variation among human and great ape genomes determined by array comparative genomic hybridization Genome Res 2003 13 3 347 357 10.1101/gr.1003303 12618365 PMC430292

[B19] Newman TL Tuzun E Morrison VA Hayden KE Ventura M McGrath SD Rocchi M Eichler EE A genome-wide survey of structural variation between human and chimpanzee Genome Research 2005 15 10 1344 1356 10.1101/gr.4338005 16169929 PMC1240076

[B20] Samonte RV Eichler EE Segmental duplications and the evolution of the primate genome Nat Rev Genet 2002 3 1 65 72 10.1038/nrg705 11823792

[B21] Ladjali-Mohammedi K Bitgood JJ Tixier-Boichard M Ponce De Leon FA International system for standardized avian karyotypes (ISSAK): standardized banded karyotypes of the domestic fowl (Gallus domesticus) Cytogenet Cell Genet 1999 86 3–4 271 276 10.1159/000015318 10575225

[B22] Chowdhary BP Raudsepp T HSA4 and GGA4: remarkable conservation despite 300-Myr divergence Genomics 2000 64 1 102 105 10.1006/geno.1999.6085 10708523

[B23] Kasai F Garcia C Arruga MV Ferguson-Smith MA Chromosome homology between chicken (Gallus gallus domesticus) and the red-legged partridge (Alectoris rufa); evidence of the occurrence of a neocentromere during evolution Cytogenet Genome Res 2003 102 1–4 326 330 10.1159/000075770 14970724

[B24] Galkina S Deryusheva S Fillon V Vignal A Crooijmans R Groenen M Rodionov A Gaginskaya E FISH on avian lampbrush chromosomes produces higher resolution gene mapping Genetica 2006 128 1–3 241 251 10.1007/s10709-005-5776-7 17028954

[B25] Musa HH Li BC Chen GH Lanyasunya TP Xu Q Bao WB Karyotype and Banding Patterns of Chicken Breeds International Journal of Poultry Science 2005 4 10 741 744 10.3923/ijps.2005.741.744

[B26] Douaud M Feve K Gerus M Fillon V Bardes S Gourichon D Dawson DA Hanotte O Burke T Vignoles F Addition of the microchromosome GGA25 to the chicken genome sequence assembly through radiation hybrid and genetic mapping BMC Genomics 2008 9 129 10.1186/1471-2164-9-129 18366813 PMC2275740

[B27] Kayang BB Fillon V Inoue-Murayama M Miwa M Leroux S Feve K Monvoisin JL Pitel F Vignoles M Mouilhayrat C Beaumont C Ito S Minvielle F Vignal A Integrated maps in quail (Coturnix japonica) confirm the high degree of synteny conservation with chicken (Gallus gallus) despite 35 million years of divergence BMC Genomics 2006 7 1 101 10.1186/1471-2164-7-101 16669996 PMC1534036

[B28] Bed'Hom B Coullin P Guillier-Gencik Z Moulin S Bernheim A Volobouev V Characterization of the atypical karyotype of the black-winged kite Elanus caeruleus (Falconiformes: Accipitridae) by means of classical and molecular cytogenetic techniques Chromosome Res 2003 11 4 335 343 10.1023/A:1024091923939 12906130

[B29] Nanda I Karl E Griffin DK Schartl M Schmid M Chromosome repatterning in three representative parrots (Psittaciformes) inferred from comparative chromosome painting Cytogenet Genome Res 2007 117 1–4 43 53 10.1159/000103164 17675844

[B30] van Tuinen M Hedges SB Calibration of avian molecular clocks Mol Biol Evol 2001 18 2 206 213 11158379 10.1093/oxfordjournals.molbev.a003794

[B31] Perry GH Tchinda J McGrath SD Zhang J Picker SR Caceres AM Iafrate AJ Tyler-Smith C Scherer SW Eichler EE Stone AC Lee C Hotspots for copy number variation in chimpanzees and humans Proc Natl Acad Sci USA 2006 103 21 8006 8011 10.1073/pnas.0602318103 16702545 PMC1472420

[B32] Steiper ME Young NM Primate molecular divergence dates Mol Phylogenet Evol 2006 41 2 384 394 10.1016/j.ympev.2006.05.021 16815047

[B33] Moon DA Veniamin SM Parks-Dely JA Magor KE The MHC of the duck (Anas platyrhynchos) contains five differentially expressed class I genes J Immunol 2005 175 10 6702 6712 16272326 10.4049/jimmunol.175.10.6702

[B34] Ahlroth MK Kola EH Ewald D Masabanda J Sazanov A Fries R Kulomaa MS Characterization and chromosomal localization of the chicken avidin gene family Anim Genet 2000 31 6 367 375 10.1046/j.1365-2052.2000.00681.x 11167523

[B35] Griffin DK Haberman F Masabanda J O'Brien P Bagga M Sazanov A Smith J Burt DW Ferguson-Smith M Wienberg J Micro- and macrochromosome paints generated by flow cytometry and microdissection: tools for mapping the chicken genome Cytogenet Cell Genet 1999 87 3–4 278 281 10.1159/000015449 10702695

[B36] Crooijmans RP Vrebalov J Dijkhof RJ Poel JJ van der Groenen MA Two-dimensional screening of the Wageningen chicken BAC library Mamm Genome 2000 11 5 360 363 10.1007/s003350010068 10790534

[B37] Groenen MA Cheng HH Bumstead N Benkel BF Briles WE Burke T Burt DW Crittenden LB Dodgson J Hillel J Lamont S de Leon AP Soller M Takahashi H Vignal A A consensus linkage map of the chicken genome Genome Res 2000 10 1 137 147 10645958 10.1101/gr.10.1.137 PMC310508

[B38] Lichter P Tang CJ Call K Hermanson G Evans GA Housman D Ward DC High-resolution mapping of human chromosome 11 by in situ hybridization with cosmid clones Science 1990 247 4938 64 69 10.1126/science.2294592 2294592

[B39] Abramoff MD Magelhaes PJ Ram SJ Image Processing with ImageJ Biophotonics International 2004 11 7 36 42

[B40] Morris WB Stephenson JE Robertson LB Turner K Brown H Ioannou D Tempest HG Skinner BM Griffin DK Practicable approaches to facilitate rapid and accurate molecular cytogenetic mapping in birds and mammals Cytogenet Genome Res 2007 117 1–4 36 42 10.1159/000103163 17675843

[B41] Workman C Jensen LJ Jarmer H Berka R Gautier L Nielser HB Saxild HH Nielsen C Brunak S Knudsen S A new non-linear normalization method for reducing variability in DNA microarray experiments Genome Biol 2002 3 9 10.1186/gb-2002-3-9-research0048 12225587 PMC126873

[B42] Tukey J Olkin E A survey of sampling from contaminated distributions Contributions to Probability and Statistics 1960 Stanford University Press 448 485

[B43] Olshen AB Venkatraman ES Lucito R Wigler M Circular binary segmentation for the analysis of array-based DNA copy number data Biostatistics 2004 5 4 557 572 10.1093/biostatistics/kxh008 15475419

