# Supplementary material for: Comparative genomics in chicken and Pekin duck using FISH mapping and microarray analysis
Source: BMC Genomics. 2009 Aug 5;10:357. doi: 10.1186/1471-2164-10-357 (PMC2907691; doi:10.1186/1471-2164-10-357)
Supplement: Additional file 1 — Chicken (Gallus gallus; GGA) BACs successfully hybridized to duck (Anas platyrhynchos; APL) macrochromosomes. BACs with these markers successfully hybridized to duck chromosomes. FLpter represents Fractional Length from the p terminus[38]; SD represents standard deviation. For reference, our previous data on turkey (Meleagris gallopavo, MGA) [2] is also included. [file 1471-2164-10-357-S1.pdf]

Additional file 1: Chicken (*Gallus gallus*; GGA) BACs successfully hybridized to duck (*Anas platyrhynchos*; APL) macrochromosomes. BACs with these markers successfully hybridized to duck chromosomes. FLpter represents Fractional Length from the p terminus [38]; SD represents standard deviation. For reference, our previous data on turkey (*Meleagris gallopavo*, MGA) [2] is also included for BACs shared by both datasets.

GGA BAC clone	GGA chromosome	Marker	GGA FLpter	GGA FLpter SD	GGA metaphases	APL chromosome	APL FLpter	APL FLpter SD	APL metaphases	MGA chromosome	MGA FLpter
WAG24H22	GGA1p	LEI0068	0.23	0.03	9	APL1p	0.24	0.02	8		
WAG30B21	GGA1p	LEI0194	0.24	0.03	8	APL1p	0.14	0.03	10	MGA1p	0.12
WAG36C14	GGA1p		0.24	0.03	6	APL1p	0.19	0.02	4		
WAG7C9	GGA1p		0.32	0.03	6	APL1q	0.40	0.02	9		
WAG38J21	GGA1p		0.38	0.03	7	APL1p	0.36	0.06	7		
WAG13K4	GGA1p		0.42	0.03	9	APL1q	0.42	0.03	6		
WAG25G16	GGA1q	LEI0101	0.47	0.03	4	APL1q	0.47	0.03	8		

**GGA BAC clone**	**GGA chromosome**	**Marker**	**GGA FLpter**	**GGA FLpter SD**	**GGA metaphases**	**APL chromosome**	**APL FLpter**	**APL FLpter SD**	**APL metaphases**	**MGA chromosome**	**MGA FLpter**
WAG39B16	GGA1q	MCW0068	0.48	0.03	10	APL1q	0.48	0.03	3		
WAG28J18	GGA1q	MCW0058	0.40	0.03	6	APL1q	0.76	0.03	5		
WAG97I11	GGA1q	LEI0160	0.54	0.03	8	APL1q	0.69	0.08	6		
WAG26C18	GGA1q	MCW0200	0.58	0.02	9	APL1q	0.64	0.08	6		
WAG12H11	GGA1q	LEI0091	0.63	0.04	8	APL1q	0.75	0.09	5		
WAG28I14	GGA1q	LEI0169	0.69	0.02	6	APL1q	0.68	0.03	7	MAG1q	0.65
WAG123C22	GGA1q	LEI0134	0.72	0.02	5	APL1q	0.71	0.06	6	MGA1q	0.92
WAG65E16	GGA1q	LEI0107	0.73	0.02	8	APL1q	0.73	0.05	5		
WAG107M5	GGA1q	ADL0101	0.90	0.04	8	APL1q	0.89	0.03	7		
WAG23I2	GGA2p	MCW0082	0.06	0.02	8	APL2p	0.09	0.02	8	MGA6	1.00
WAG19L16	GGA2p	ADL0309	0.21	0.03	3	APL2p	0.27	0.06	5	MGA6	0.28

GGA BAC clone	GGA chromosome	Marker	GGA FLpter	GGA FLpter SD	GGA metaphases	APL chromosome	APL FLpter	APL FLpter SD	APL metaphases	MGA chromosome	MGA FLpter
WAG40A2	GGA2p	ADL0176	0.25	0.03	6	APL2p	0.23	0.04	4		
WAG41H16	GGA2p	MCW0239	0.25	0.04	7	APL2p	0.24	0.04	11		
WAG26E13	GGA2p	MCW0131	0.25	0.03	5	APL2p	0.25	0.04	9	MGA6	0.15
WAG85M7	GGA2p	ADL0120	0.27	0.02	3	APL2p	0.27	0.06	7		
WAG82N9	GGA2p	MCW0274	0.29	0.03	3	APL2p	0.29	0.04	3		
WAG15I23	GGA2p		0.31	0.04	11	APL2p	0.41	0.04	9		
WAG40I2	GGA2p		0.34	0.03	10	APL2p	0.39	0.06	7		
WAG18L21	GGA2q		0.36	0.03	10	APL2p	0.37	0.03	9		
WAG9L1	GGA2q	LEI0129	0.42	0.03	8	APL2p	0.31	0.03	6	MGA3	0.00
WAG41G5	GGA2q		0.42	0.03	11	APL2p	0.33	0.07	8		
WAG41E24	GGA2q		0.51	0.04	10	APL2q	0.55	0.04	11		

GGA BAC clone	GGA chromosome	Marker	GGA FLpter	GGA FLpter SD	GGA metaphases	APL chromosome	APL FLpter	APL FLpter SD	APL metaphases	MGA chromosome	MGA FLpter
WAG41C2	GGA2q	LEI0147	0.67	0.03	2	APL2q	0.69	0.04	8	MGA3	0.46
WAG65M19	GGA2q	ABR0008	0.68	0.02	7	APL2q	0.67	0.05	9		
WAG26A22	GGA2q	MCW0157	0.96	0.02	7	APL2q	0.96	0.02	7		
WAG29L12	GGA3	MCW0261	0.09	0.04	7	APL3	0.12	0.04	8	MGA2	0.00
WAG36I7	GGA3	ADL0370	0.24	0.05	6	APL3	0.28	0.03	5	MGA2	0.26
WAG9P24	GGA3	MCW0127	0.55	0.04	4	APL3	0.60	0.04	2	MGA2	0.57
WAG112C24	GGA4p	ADL0317	0.05	0.02	8	APL10	0.45	0.05	6	MGA9	1.00
WAG24P2	GGA4p		0.06	0.02	9	APL10	0.80	0.09	8		
WAG23C4	GGA4p		0.06	0.03	9	APL10	0.90	0.06	5		
WAG23I6	GGA4p	ADL0203	0.12	0.02	6	APL10	0.34	0.06	4	MGA9	0.11
WAG18H15	GGA4p		0.15	0.04	10	APL10	0.24	0.08	6		

GGA BAC clone	GGA chromosome	Marker	GGA FLpter	GGA FLpter SD	GGA metaphases	APL chromosome	APL FLpter	APL FLpter SD	APL metaphases	MGA chromosome	MGA FLpter
WAG13I5	GGA4q		0.40	0.06	12	APL4	0.52	0.07	9		
WAG22J17	GGA4q		0.47	0.05	15	APL4	0.17	0.07	8		
WAG23K3	GGA4q		0.53	0.04	9	APL4	0.34	0.05	7		
WAG36E8	GGA4q		0.56	0.03	7	APL4	0.61	0.07	10		
WAG21J21	GGA4q		0.58	0.03	7	APL4	0.25	0.08	10		
WAG75K23	GGA4q		0.88	0.06	12	APL4	0.86	0.05	8		
WAG13E2	GGA4q	LEI0063	0.89	0.04	6	APL4	0.92	0.04	5	MGA4	0.94
WAG18I11	GGA4q		0.93	0.03	9	APL4	0.92	0.03	10		
WAG37H20	GGA5	MCW0263	0.24	0.06	4	APL5	0.22	0.07	4	MGA5	0.05
WAG40L16	GGA5	MCW0193	0.38	0.06	3	APL5	0.46	0.08	5	MGA5	0.55
WAG27H3	GGA5	ROS0013	0.42	0.06	8	APL5	0.51	0.06	11	MGA5	0.48

GGA BAC clone	GGA chromosome	Marker	GGA FLpter	GGA FLpter SD	GGA metaphases	APL chromosome	APL FLpter	APL FLpter SD	APL metaphases	MGA chromosome	MGA FLpter
WAG03K18	GGA5	MCW0210	0.62	0.05	7	APL5	0.54	0.03	4	MGA5	0.60
WAG89B24	GGA5	MCW0113	0.70	0.06	9	APL5	0.71	0.07	6		
WAG03O23	GGA5	ADL0166	0.81	0.08	5	APL5	0.72	0.08	5		
WAG59L17	GGA6		0.45	0.11	11	APL6	0.41	0.13	4		
WAG30C21	GGA6	ADL0040	0.49	0.07	4	APL6	0.36	0.10	4	MGA8	0.76
WAG21C4	GGA6		0.62	0.12	10	APL6	0.65	0.10	5		
WAG59A2	GGA6	ROS003	0.62	0.08	6	APL6	0.66	0.08	7	MGA8	0.92
WAG59C5	GGA6		0.80	0.07	10	APL6	0.73	0.11	10		
WAG69P21	GGA7	LEI0064	0.08	0.05	6	APL7	0.26	0.07	11	MGA7	0.10
WAG59H11	GGA7		0.17	0.07	12	APL7	0.33	0.11	8		
WAG21P13	GGA7	MCW0120	0.31	0.07	6	APL7	0.24	0.06	7	MGA7	0.29

GGA BAC clone	GGA chromosome	Marker	GGA FLpter	GGA FLpter SD	GGA metaphases	APL chromosome	APL FLpter	APL FLpter SD	APL metaphases	MGA chromosome	MGA FLpter
WAG38B23	GGA7		0.40	0.13	10	APL7	0.38	0.11	7		
WAG27C3	GGA7	MCW0201	0.44	0.07	3	APL7	0.46	0.03	2	MGA7	0.40
WAG41A15	GGA7		0.45	0.11	11	APL7	0.54	0.09	10		
WAG44J7	GGA7		0.64	0.06	4	APL7	0.59	0.08	4		
WAG15E10	GGA7		0.86	0.05	8	APL1q	0.84	0.07	9		
WAG29C17	GGA8	MCW0275	0.14	0.03	6	APL8	0.45	0.04	3	MGA10	0.50
WAG59F7	GGA8		0.29	0.09	11	APL8	0.50	0.14	8		
WAG60M16	GGA8	ADL0302	0.45	0.08	3	APL8	0.54	0.05	10	MGA10	0.51
WAG21G17	GGA8		0.61	0.10	9	APL8	0.63	0.10	6		
WAG64N5	GGA8		0.72	0.14	9	APL8	0.74	0.09	11		
WAG43P21	GGA8	ADL0301	0.75	0.05	3	APL8	0.87	0.07	4	MGA10	0.95

GGA BAC clone	GGA chromosome	Marker	GGA FLpter	GGA FLpter SD	GGA metaphases	APL chromosome	APL FLpter	APL FLpter SD	APL metaphases	MGA chromosome	MGA FLpter
WAG37E21	GGA8		0.80	0.11	9	APL8	0.87	0.06	10		
WAG40M19	GGAZ		0.08	0.04	14	APLZ	0.91	0.04	8		
WAG17D3	GGAZ		0.11	0.04	19	APLZ	0.91	0.04	8		
WAG41P6	GGAZ		0.27	0.04	10	APLZ	0.69	0.08	10		
WAG19N8	GGAZ		0.48	0.05	10	APLZ	0.56	0.06	10		
WAG20L10	GGAZ		0.74	0.05	10	APLZ	0.11	0.06	11		
WAG22P4	GGAZ		0.80	0.06	8	APLZ	0.16	0.06	9		
