# Supplementary material for: Comparative genomics in chicken and Pekin duck using FISH mapping and microarray analysis
Source: BMC Genomics. 2009 Aug 5;10:357. doi: 10.1186/1471-2164-10-357 (PMC2907691; doi:10.1186/1471-2164-10-357)
Supplement: Additional file 2 — BACs on duck (Anas platyrhynchos; APL) microchromosomes. BACs with the named markers successfully hybridized to duck chromosomes. Duck chromosome number after APL10 was assigned as chicken (GGA) chromosome number plus one. The orthologous turkey (Meleagris gallopavo, MGA) chromosome (from [2]) is also listed. [file 1471-2164-10-357-S2.pdf]

**Additional file 2**: BACs on duck (*Anas platyrhynchos*; APL) microchromosomes. BACs with the named markers successfully hybridized to duck chromosomes. Duck chromosome number after APL10 was assigned as chicken (GGA) chromosome number plus one. The orthologous turkey (*Meleagris gallopavo*, MGA) chromosome (from [2]) is also listed.

| GGA chromosome | GGA BAC Clone | GGA Marker | APL chromosome | MGA chromosome |
|---|---|---|---|---|
| GGA9 | WAG9J23 | ROS0078 | APL9 | MGA11 |
| GGA9 | WAG27D19 | ADL0191 | APL9 | MGA11 |
| GGA9 | WAG14A21 | MCW0134 | APL9 | MGA11 |
| GGA10 | WAG8K20 | B2Msts1 | APL 11 | MGA12 |
| GGA10 | WAG15I8 | ADL0272 | APL11 | MGA12 |
| GGA10 | WAG33O18 | CYP19 | APL11 | |
| GGA10 | WAG18I3 | MCW0035 | APL 11 | MGA12 |
| GGA10 | WAG8G10 | MCW0132 | APL11 | MGA12 |
| GGA10 | WAG35L19 | MCW0003 | APL11 | MGA12 |
| GGA11 | WAG54E23 | ADL0308 | APL12 | MGA13 |
| GGA12 | WAG4I21 | SCW0019 | APL13 | MGA14 |
| GGA12 | WAG47D10 | LEI0099 | APL13 | MGA14 |
| GGA13 | WAG14G12 | MCW0244 | APL14 | MGA15 |
| GGA13 | WAG18H2 | MCW0213 | APL 14 | MGA15 |
| GGA13 | WAG27P20 | MSX2sts1 | APL 14 | MGA15 |
| GGA13 | WAG25H18 | CAMLGsts1 | APL14 | MGA15 |
| GGA13 | WAG8N24 | POU4F3sts1 | APL14 | MGA15 |
| GGA14 | WAG43B20 | MCW0296 | APL15 | MGA16 |
| GGA14 | WAG26J16 | GCT0908 | APL15 | MGA16 |

| GGA chromosome | GGA BAC Clone | GGA Marker | APL chromosome | MGA chromosome |
|---|---|---|---|---|
| GGA14 | WAG53L23 | ADL0200 | APL15 | MGA16 |
| GGA15 | WAG10L1 | LEI0083 | APL16 | MGA17 |
| GGA15 | WAG109B14 | MCW0031 | APL16 | MGA17 |
| GGA15 | WAG17C11 | LEI0120 | APL16 | MGA17 |
| GGA15 | WAG62P3 | SFPQ | APL16 | |
| GGA15 | WAG129C21 | ABR0070 | APL16 | MGA17 |
| GGA15 | WAG7G1 | MCW0211 | APL16 | MGA17 |
| GGA15 | WAG30E18 | MCW0080 | APL16 | MGA17 |
| GGA15 | WAG93I1 | CRYBA4sts1 | APL16 | MGA17 |
| GGA16 | WAG65G9 | MCW0371 | APL17 | MGA18 |
| GGA16 | WAG65G9 | LEI0258 | APL17 | MGA18 |
| GGA17 | WAG40G23 | ADL0149 | APL18 | MGA19 |
| GGA17 | WAG13I13 | MCW0151 | APL 18 | MGA19 |
| GGA18 | WAG0O20 | ADL0184 | APL 19 | MGA20 |
| GGA18 | WAG19B13 | MCW0045 | APL 19 | MGA20 |
| GGA18 | WAG122G20 | HUJ0010 | APL19 | MGA20 |
| GGA18 | WAG27I7 | ROS0022 | APL19 | MGA20 |
| GGA18 | WAG34F23 | ADL0290 | APL19 | MGA20 |
| GGA18 | WAG1D2 | MCW0219 | APL19 | MGA20 |
| GGA18 | WAG14H23 | ROS0027 | APL19 | MGA20 |
| GGA19 | - | CTG1704 | APL20 | |
| GGA19 | - | CTG7040 | APL20 | |

| GGA chromosome | GGA BAC Clone | GGA Marker | APL chromosome | MGA chromosome |
|---|---|---|---|---|
| GGA19 | WAG20G24 | MCW0256 | APL 20 | MGA21 |
| GGA19 | WAG55M22 | SCW0024 | APL20 | MGA21 |
| GGA20 | - | GTC20050 | APL21 | MGA22 |
| GGA20 | WAG120F16 | MCW0119 | APL21 | MGA22 |
| GGA20 | WAG22L3 | ADL0193 | APL21 | MGA22 |
| GGA20 | WAG84E10 | ADL0034 | APL21 | MGA22 |
| GGA20 | WAG10A11 | FZFsts1 | APL21 | MGA22 |
| GGA21 | WAG24C7 | AGRIN | APL22 | |
| GGA21 | WAG21B10 | PLODsts1 | APL22 | |
| GGA22 | WAG32L22 | ROS0073 | APL23 | |
| GGA22 | - | TVSB3sts1 | APL23 | |
| GGA23 | - | CTG1080 | APL24 | |
| GGA23 | - | CTG1100 | APL24 | |
| GGA23 | WAG25H8 | ADL0262 | APL24 | MGA25 |
| GGA23 | WAG29E23 | LEI0102 | APL24 | MGA25 |
| GGA23 | WAG46K8 | MCW0165 | APL24 | MGA25 |
| GGA23 | WAG10D11 | ADL0289 | APL24 | MGA25 |
| GGA23 | WAG96F24 | LEI0339 | APL24 | MGA25 |
| GGA23 | WAG28L18 | LEI0090 | APL24 | MGA25 |
| GGA24 | WAG13J20 | ROS0113A | APL25 | MGA26 |
| GGA24 | WAG8L4 | ROS0123 | APL25 | MGA26 |
| GGA24 | WAG20E8 | APOA1sts | APL25 | MGA26 |

| GGA chromosome | GGA BAC Clone | GGA Marker | APL chromosome | MGA chromosome |
|---|---|---|---|---|
| GGA24 | WAG32F12 | LEI0069 | APL25 | MGA26 |
| GGA26 | - | CTG1120 | APL27 | |
| GGA26 | WAG34F14 | ABR0330 | APL27 | |
| GGA26 | WAG5G11 | MCW0286 | APL27 | MGA28 |
| GGA26 | WAG28I1 | MCW0069 | APL27 | MGA28 |
| GGA26 | WAG96B10 | LEI0074 | APL27 | |
| GGA27 | WAG9E8 | MCW0146 | APL28 | MGA29 |
| GGA27 | WAG8C15 | MCW0328 | APL28 | MGA29 |
| GGA27 | WAG3A13 | GCT0022 | APL28 | MGA29 |
| GGA28 | - | CTG0070 | APL29 | |
| GGA28 | WAG36G5 | LEI0135 | APL29 | MGA30 |
| GGA28 | WAG24J22 | ABR0341 | APL29 | MGA30 |
| GGA28 | WAG102C19 | ABR0032 | APL29 | |
| GGA28 | WAG95C14 | ABR0054 | APL29 | |
| GGA28 | WAG75L5 | GCT0902 | APL29 | |
| GGA28 | WAG32P6 | ADL0299 | APL29 | |
| GGA28 | WAG29E8 | LEI0067A | APL29 | MGA30 |
